# Predicting Outcome in an Intensive Outpatient PTSD Treatment Program Using Daily Measures

**DOI:** 10.3390/jcm10184152

**Published:** 2021-09-15

**Authors:** Valentijn V. P. Alting van Geusau, Jeroen D. Mulder, Suzy J. M. A. Matthijssen

**Affiliations:** 1 Altrecht Academic Anxiety Center, Altrecht GGz, 3524 SH Utrecht, The Netherlands; valentijnavgeusau@gmail.com; 2 Department of Clinical Psychology, Utrecht University, 3584 CS Utrecht, The Netherlands; 3 Department of Methodology and Statistcs, Faculty of Social and Behavioural Sciences, Utrecht University, 3584 CS Utrecht, The Netherlands; j.d.mulder@uu.nl

**Keywords:** intensive treatment program, PTSD, EMDR, prolonged exposure, daily measured symptoms, outcome, predictor

## Abstract

It is useful to investigate factors that could predict treatment outcomes for PTSD. The current study aims to investigate the relationship between daily measured PTSD symptoms during an intensive six-day treatment program and overall post-treatment outcomes. The treatment program combines eye movement desensitization with reprocessing and prolonged exposure, as well as physical activity and psychoeducation. It was expected that for the entire duration of treatment, as well as the first half of the treatment, a greater decline in daily PTSD symptoms would be a predictor for a greater decline in PTSD symptoms at a four-week follow-up. Data from 109 PTSD-patients (87.2% female, mean age = 36.9, *SD* = 11.5) were used. PTSD symptoms were measured with the CAPS-5 and the self-reported PTSD checklist for DSM-5 (PCL-5). Daily PTSD symptoms were measured with an abbreviated version of the PCL-5 (8-item PCL). Latent growth curve models were used to describe changes in daily PTSD symptoms and predict treatment outcome. Results show that a greater decline in daily PTSD symptoms measured by the 8-item PCL predicts better treatment outcome (CAPS-5 and PCL-5), but that a patient’s PTSD symptoms on the first day of treatment has no predictive effect. A decline in PTSD symptoms only during the first half of treatment was also found to predict treatment outcomes. Future research should be focused on replicating the results of the current study.

## 1. Introduction

Post-traumatic Stress Disorder (PTSD) is a stress-related disorder that one can develop after being exposed to one or more traumatic events [1]. The lifetime prevalence of PTSD is around 7.4–8% [2,3]. According to multidisciplinary guidelines, there are several evidence-based treatments for PTSD [4,5]. Among these are Eye Movement Desensitization and Reprocessing (EMDR), Trauma-Focused Cognitive Behavioral Therapy (TF-CBT), Prolonged Exposure (PE), and Cognitive Processing Therapy (CPT) which all show good effect sizes in reducing PTSD symptoms [6]. EMDR therapy seems to be the most cost-effective treatment [7].

Although there are effective trauma treatments, drop-out rates are often high. In a meta-analysis by [8], an average drop-out rate of 18% was found among active treatments in clinical trials for PTSD but drop-out rates as high as 54% are reported in some studies [9]. Furthermore, of all the patients who complete treatment, 30–50% still show symptoms [10]. Therefore, there is much room for improvement. A first step would be to find out who is likely to benefit from treatment and who is not, and to see if treatment success can already be predicted in an early phase. If so, practitioners may decide to scale up or alter treatment during early stages of treatment, which would prevent patients from undergoing treatment that is predicted to have little effect in the long term. 

In some studies, factors related to treatment outcome for psychotherapeutic interventions for PTSD were identified, including comorbidity, cognitive dimensions, suicide risk and characteristics of the patient such as gender [11,12,13]. Results of a study investigating predictors of treatment outcome and drop-out in two samples of PTSD patients who were treated with PE, showed that higher PTSD symptom scores at pre-treatment were correlated with more PTSD symptoms at post-treatment and at follow-up [14]. Another study found that lower pre-treatment clinician-rated PTSD symptoms were associated with better treatment outcomes, whereas higher baseline self-rated PTSD symptoms were associated with better treatment outcomes [15]. In one study, indications were found that benzodiazepine use was related to worse treatment outcomes, and alcohol use was related to increased drop-out rates. However, demographic variables; depression; general anxiety; personality pathology; trauma characteristics; feelings of anger, guilt, and shame; and nonspecific variables regarding therapy were not related to either treatment outcomes or dropping out [14]. The result that the use of benzodiazepines was related to worse PTSD psychotherapy outcomes has also been found in a meta-analysis [16]. Although in some studies factors were identified that were related to treatment outcomes, in other studies contradictory results were found. Hence, there are not many clear, convincing and reliable pre-treatment predictors for treatment outcomes, a result that has also been found in other studies [15,17]. However, how about predictors during treatment? Is it possible to predict treatment outcomes during early stages of treatment? 

Several factors during treatment have been shown to predict treatment outcome. A strong therapeutic alliance has been linked to better treatment outcome in psychotherapeutic interventions [18,19]. Between-session habituation has been identified as a predictor for treatment outcomes in PE treatment programs, with patients who showed more between-session habituation being more likely to show better treatment outcomes [20,21]. It has also been shown that trauma-related belief change predicted subsequent PTSD-symptom change in PE [20]. Higher fear activation during the first session of PE, as measured with subjective units of distress (SUDs) and facial expression, was found to be correlated with better treatment outcome [22]. Higher emotional engagement during PE in the first session, as measured with SUDs, predicted better treatment outcomes [23]. For EMDR, it was found that lower SUD scores at the end of the first session predicted better treatment results [24]. 

In identifying predictors of treatment outcome, one could argue that it is clinically relevant to identify treatment response in an early stage in order to be able to adjust treatment strategies when deemed necessary. In a study examining PE effects for PTSD symptoms for veterans of the war in Iraq, the greatest reduction in symptoms was found in the first five sessions [25]. In another study, comparing EMDR to brief eclectic psychotherapy, it was also found that the largest reduction in PTSD symptoms was achieved in the first five sessions in the EMDR condition [26]. However, only a few researchers have studied whether the early response progress predicts post-treatment outcomes. For example, one study found that PTSD patients receiving PE or CPT who did not improve much after the first eight sessions were not likely to improve much subsequently [27]. In another study it was found that the probability of achieving meaningful symptom amelioration decreased after every session for patients receiving CPT, indicating that patients who show little PTSD-symptom change during early stages of treatment are likely to show worse overall treatment outcomes [28].

The present study aims to respond to the limited evidence for early treatment response as a predictor for treatment success. Treatment for PTSD is commonly delivered once or twice a week over the course of several months. Since PTSD interferes with social and occupational functioning [29], it is desirable for patients to make rapid progress. Several intensive treatment programs have been set up, with good results and significantly lower drop-out rates of below 10% [30]. The current study aims to determine the predictive value of treatment response on treatment outcome in such an intensive treatment program, which consists of two weeks of treatment for three consecutive days each. Patients receive three hours of trauma therapy (PE and EMDR), one hour of physical activity and one hour of psychoeducation every day. The results of a meta-analysis showed that adding physical activity to usual care improved the health of PTSD patients and was effective in decreasing PTSD symptoms [31]. A combination of PE, EMDR, physical activity and psychoeducation in an inpatient intensive treatment program was found to be effective in reducing PTSD symptoms [32].

The goal of the present study is to investigate whether the change in PTSD symptomatology during the current intensive treatment program, as measured by daily self-reports, can predict treatment outcomes. It is expected that a greater decline in PTSD symptoms during the total treatment program is a predictor for greater decrease in PTSD symptoms after treatment completion. In addition, it is expected that a greater decline in PTSD symptoms during the first half of the treatment program is a predictor for overall treatment outcome as well. It is expected that patients who do not show much symptom reduction during the first sessions will show worse overall treatment outcomes.

## 2. Materials and Methods

### 2.1. Participants

The current study used a self-select sample comprised of 109 PTSD patients who attended the program between April 2018 and November 2019. The mean age was 36.9 (*SD* = 11.5) ranging from 20 to 64 years; 14 identified as male (12.8%), 95 as female (87.2%). The trauma types of patients varied (e.g., sexual abuse, physical abuse and accidents). Inclusion criteria were: having a PTSD diagnosis according to the DSM-5 [1], having experienced multiple traumatization, no alcohol or drug use during treatment, no acute suicidality risk, sufficient proficiency in the Dutch language, the absence of comorbid psychiatric disorders that would seriously interfere with treatment, and no (or in exceptional cases, minimal) use of sedating medication during treatment (exception, e.g., patients prone to mania when they would be deprived of sleep were allowed to continue sleeping medication). 

### 2.2. Procedure

The intensive trauma treatment program was provided at the Altrecht Academic Anxiety Center, a center specialized in the treatment of severe anxiety disorders, OCD and trauma-related disorders in Utrecht, the Netherlands. Prior to starting treatment, participants were screened on diagnoses and inclusion criteria, and an individual treatment plan was made. Participants were asked to select the six subjectively most disturbing traumatic memories for treatment. Each day, one memory would be treated. 

### 2.3. Treatment

The treatment program consisted of two consecutive weeks with treatment—delivered in an outpatient setting—provided on Tuesday, Wednesday and Thursday. At the start of the first treatment day in the first week and at the end of the last treatment day in the second week, patients filled out measurements. Each treatment day then had the same outline (see Table 1). Treatment consisted of two evidence-based treatments for PTSD: PE and EMDR. For the PE sessions, a slightly modified version of the PE protocol was used [33]. Patients did not make audio recordings to listen to as homework in between sessions. EMDR was delivered according to standard protocol [34,35]. The combination of these treatments was used because PE and EMDR supposedly differ in underlying working mechanism and for this reason could possibly complement each other in treatment effect. It has also been found that these treatments can be successfully combined [36]. Patients received PE in the morning and EMDR in the afternoon. It has been shown that this sequence resulted in better treatment outcome than the reversed sequence [36]. Treatment was delivered with therapist rotation to ensure patients were treated by many different therapists, and the therapists had daily multidisciplinary meetings in between sessions to ensure treatment was given according to protocol. The therapist rotation approach was used because it is suggested that this approach possibly leads to better implementation of PTSD treatments because of the decrease of therapist drift and reduced negative concerns by therapists due to the shared responsibility [37]. In between PE and EMDR sessions, patients conducted physical activity: either trauma-sensitive yoga [38,39], walking or jogging, or physical exercises. 

### 2.4. Measurements

**Figure 1:**
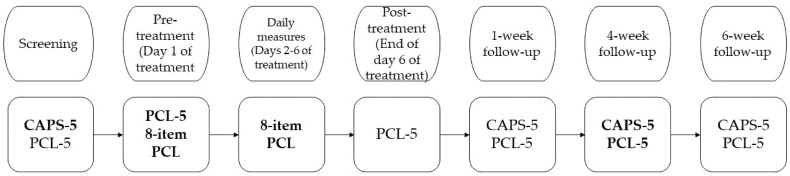
Measuring moments. The pre-treatment and daily measures were conducted at the start of the day before actual treatment was delivered. Not all measuring moments were used in the current study, the measurements in bold were the ones used.

Multiple instruments were used to assess a variety of symptoms. For the current study, only study-related measures will be discussed. Figure 1 contains an overview of the timing of these measures throughout the study.

- The Dutch version of the Clinician Administered PTSD Scale for DSM-5 (CAPS-5) assesses the frequency and intensity of the 20 DSM-5 PTSD symptoms [40,41]. Severity scores were computed as a sum score of the 20 symptom-specific severity scores, ranging from 0–80. The CAPS-5 was used for evaluating the existence of a PTSD diagnosis and measuring the change in PTSD symptoms. It has adequate validity and reliability [42].
- The Dutch version of the PTSD checklist for DSM-5 (PCL-5) is a 20-item self-report questionnaire intended to measure PTSD symptomatology with scores ranging from 0–80 [43,44]. It was administered to participants to measure the difference in PTSD symptomatology before and after treatment, and shows strong validity and reliability [45].
- An abbreviated 8-item version of the Dutch PCL-5 (from here onwards referred to as the 8-item PCL) was used to monitor the daily PTSD symptoms during treatment. This self-report instrument consists of 8 of the original 20 questions from the PCL-5 with scores ranging from 0–32. The 8-item PCL strongly correlated with the complete PCL-5 and has been recommended for use to monitor treatment progress [46]. For interpretive data on the 8-item PCL, readers are referred to Price et al. [46].

### 2.5. Data Analysis

Latent growth curve models (LGCM) [47] were fitted to the daily PTSD measurement (8-item PCL) to capture the change in PTSD symptomatology of participants during the treatment program. In LGCM, this change is described by latent factors which we refer to as growth factors. The models were then extended by including two follow-up treatment outcome variables, which were operationalized as the difference in CAPS-5 (ΔC) scores before and four weeks after the treatment, and the difference in PCL-5 (ΔP) scores before and four weeks after the treatment. These outcome variables were regressed on the growth factors to investigate to what degree progress during treatment (as described by the growth factors) could predict change in PTSD symptomatology four weeks after treatment (i.e., the follow-up treatment outcomes).

**Figure 2:**
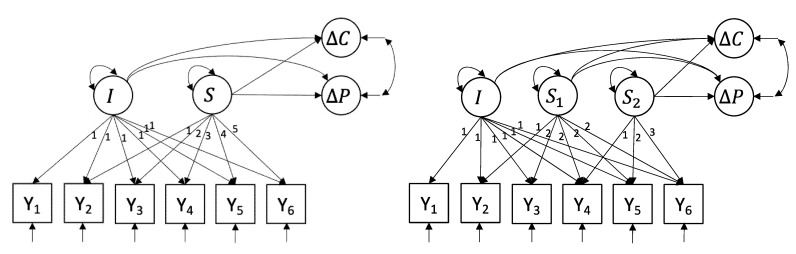
The linear LGCM (**left**) and the piecewise LGCM (**right**). Y = daily measurement of PTSD symptomatology using the 8-item PCL; I = intercept growth factor; *S* = linear slope growth factor; *S*_1_ = linear slope growth factor week 1; *S*_2_ = linear slope growth factor week 2; ΔC = difference in CAPS-5 before and after treatment; ΔP = difference in PCL-5 before and after treatment.

As LGCM are flexible models that can differ in the number and type of growth factors used to capture progress during treatment, multiple candidate LGCM (i.e., linear, quadratic and piecewise LGCMs) were compared with respect to how well the follow-up treatment outcomes could be predicted. Full details on the different candidate models considered, their prediction performance and model fit can be found in the online Supplementary Materials at https://jeroendmulder.github.io/predicting-PTSD-using-LGCM (accessed on 13 September 2021). Here, we focus on the both the basic linear LGCM (L-LGCM; see left panel Figure 2), as it is the most parsimonious model while retaining good prediction performance, and the piece-wise linear LGCM (P-LGCM; see right panel Figure 2), because it is interesting from a clinical point of view, as explained below. Analyses were performed using the *lavaan* package version 0.6–7 [48] in R version 3.6.1 [49]. Missing data were handled using full information maximum likelihood, such that all available datapoints were used in the analyses (i.e., no patients were listwise deleted).

The L-LGCM uses two growth factors, an intercept *I* and a linear slope *S*, to capture initial PTSD symptomatology of participants at start of treatment, and linear change in symptomatology over all six daily measures, respectively. These factors then predict the follow-up treatment outcome variables ΔC and ΔP. The P-LGCM extends the L-LGCM by including a second linear slope factor *S*_2_: The first linear slope factor *S*_1_ then captures linear change in symptomatology during the first week of treatment (the first three daily measures), whereas the second linear slope factor captures linear change in symptomatology during the second week (the last three daily measures). All three growth factors then predict the follow-up treatment outcome variables. This model is interesting from a clinical perspective as it can estimate the predictive power of the first two full days of treatment (measurements were taken at the start of day 1–3) and days three, four and five of treatment (measurements of day 4–6) separately.

## 3. Results

**Figure 3:**
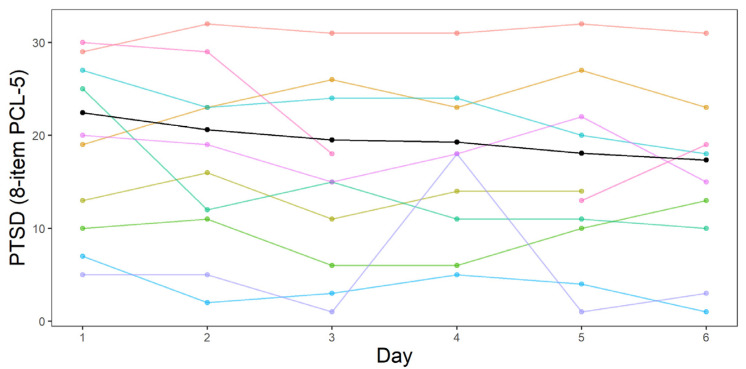
PTSD symptomatology during treatment, as measured with the 8-item PCL, of a sample of 10 participants (see the online Supplementary Materials for the complete sample). The *y*-axis represents total score on the 8-item PCL. The *x*-axis represents treatment day. The black line represents the observed mean PTSD symptomatology over time, whereas the colored lines represent individual trajectories.

In this section, we present and visualize the data and discuss the results of the L-LGCM and the P-LGCM. Descriptive statistics for the follow-up treatment outcome variables, as well as the 8-item PCL daily measures, can be found in Table 2. At screening, patients had an average CAPS-5 score of *M* = 43.10 (*SD* = 9.93), and at the pre-treatment measurement occasions, patients had an average PCL-5 score of *M* = 55.08 (*SD* = 11.04). Figure 3 depicts the daily PTSD measurements throughout treatment for a sample of 10 participants (to avoid overplotting, these participants were selected here to represent the large range of PTSD symptoms in the sample throughout the treatment). The solid black line represents the average change in PTSD symptoms over the entire sample. An interactive plot containing the daily PTSD measurements from the entire sample can be found in the online Supplementary Materials. Figure 3 shows that there are large differences in both the initial PTSD symptoms of participants (as can also be seen by the standard deviation of the daily measurements in Table 1), as well as how much participants change and the form of this change during the treatment program. Four weeks after completion of the treatment program, 45 participants (41.3%) still met the criteria for PTSD according to the CAPS-5, 48 participants (44.0%) did not meet the criteria for PTSD anymore, and 16 (14.7%) cases were missing (i.e., the participants did not show up for the four-week follow-up measurement or they showed up too late). Patients had an average CAPS-5 score of *M* = 25.82 (*SD* = 17.00) and an average PCL-5 score of *M* = 34.02 (*SD* = 20.08) at follow-up. By taking the difference in CAPS-5 and PCL-5 before and after treatment, we found a mean follow-up treatment outcome of *M* = −17.29 (*SD* = 15.47) on the CAPS-5, and *M* = −22.05 (*SD* = 19.61) for the PCL-5. 

### 3.1. The Linear LGCM (L-LGCM)

The L-LGCM resulted in good model fit: $\chi^{2}$ (25) = 35.98, *p* = 0.072, root-mean-square error of approximation (RMSEA) = 0.06, comparative fit index (CFI) = 0.99, and Tucker-Lewis index (TLI) = 0.98. The mean of the intercept factor was estimated at 21.94 (*SE* = 0.56, *p* < 0.001) with a variance of 29.45 (*SE* = 4.56, *p* < 0.001), and the mean of the linear slope factor was estimated to be −0.96 (*SE* = 0.12, *p* < 0.001) with a variance of 1.15 (*SE* = 0.23, *p* < 0.001). This implies that on average, patients started the treatment program with a PTSD score of 21.94 on the 8-item PCL scale, which decreased linearly each day by 0.96 points. However, the variances of the growth factors imply that there are large differences in the starting point and change during the treatment program between patients.

When predicting the follow-up treatment outcome ΔP from the growth components, we found a nonsignificant regression coefficient (unstandardized) for the intercept (*B* = 0.20, *SE* = 0.30, *p* = 0.492), but a significant coefficient for the slope (*B* = 15.44, *SE* = 1.81, *p* < 0.001). Together, the intercept and slope result in an *R*^2^ of 0.69. This implies that the linear slope in the L-LGCM is a significant predictor of change in PTSD symptomatology on the PCL-5 scale from pre-treatment to four-week follow-up, explaining approximately 69% of the variance. Patients who show a one-point-per-day greater decrease in their PTSD symptomatology during treatment are predicted to have a 15.44point greater decrease in PTSD symptoms on the PCL-5 scale from pre-treatment to four-week follow-up. The symptomatology at the start of the treatment program does not provide any predictive information about ΔP. For follow-up treatment outcomes measured using the CAPS-5, ΔC, we again found a nonsignificant regression coefficient (unstandardized) for the intercept (*B* = 0.25, *SE* = 0.24, *p* = 0.298) but a significant coefficient for the slope (*B* = 11.76, *SE* = 1.47, *p* < 0.001). Together, the intercept and slope produce an *R*^2^ of 0.66. This suggests that the intercept is not predictive of follow-up treatment outcome on the CAPS-5 scale, but participants with 1-point greater decrease in PTSD symptomatology per day are predicted to have in a 11.76 greater decrease in ΔC. Both the intercept and slope explain approximately 66% of the variance. 

### 3.2. The Piecewise LGCM (P-LGCM)

The P-LGCM resulted in adequate model fit: $\chi^{2}$ (21) = 33.99, *p* = 0.036, RMSEA = 0.08, CFI = 0.98 and TLI = 0.98. The mean of the intercept was estimated at 22.18 (*SE* = 0.57, *p* < 0.001) with a variance of 28.04 (*SE* = 4.45, *p* < 0.001), the mean of the slope across day one to three was −1.20 (*SE* = 0.26, *p* < 0.001) with a variance of 3.51 (*SE* = 0.95, *p* < 0.001), and the slope across day 4 to 6 was −0.85 (*SE* = 0.16, *p* < 0.001) with a variance of 1.36 (*SE* = 0.37, *p* < 0.001). This implies that on average, patients started treatment with a score of 22.18 on the 8-item PCL. Over the first two full treatment days, PTSD symptomatology decreased on average with approximately 1.20 points per day, whereas in the second week the symptomatology decreased daily with approximately 0.85 points. 

The follow-up treatment outcome variables regressed on the growth components. For ΔP we found an unstandardized regression coefficient of *B* = 0.12 for the intercept (*SE* = 0.32, *p* = 0.693), *B* = 6.47 for the first slope (*SE* = 1.41, *p* < 0.001) and *B* = 8.83 for the second slope (*SE* = 2.03, *p* < 0.001). These results indicate that both changes in PTSD symptomatology in the first week as well as changes in the second week are significant predictors for follow-up treatment outcomes on the PCL-5 scale. We found an *R*^2^ of 0.67, meaning that 67% of the outcome is explained by all three growth components combined. Next, we inspected the standardized regression coefficients to compare which predictor (either the change in PTSD symptomatology the first week or the second week) has greater predictive power. For the first slope we found β = 0.62 (*SE* = 0.11, *p* < 0.001) and for the second slope β = 0.53 (*SE* = 0.11, *p* < 0.001). We therefore concluded that both the first and the second slope are useful in predicting follow-up treatment outcomes (PCL-5), but relatively speaking, the change in PTSD symptomatology during the first week holds more predictive power than the change in symptoms during the second week. This is also reflected in the amount of explained variance when using only the first slope to predict the outcome: When we omit the slope from the second week from the model, a significant amount of variance (*R*^2^ = 0.33) is still explained using the intercept and the first slope. However, this is 34 percentage points less compared to using all three growth factors. 

For follow-up treatment outcomes on the CAPS-5 scale, we again found a nonsignificant regression coefficient (unstandardized) for the intercept (*B* = 0.28, *SE* = 0.26, *p* = 0.270) but a significant coefficient for the first slope (*B* = 4.17, *SE* = 1.09, *p* < 0.001) and the second slope (*B* = 7.73, *SE* = 1.76, *p* < 0.001). The growth components combined explain approximately 63% of variance in the outcome. Looking at the standardized effects, we found β = 0.52 (*SE* = 0.12, *p* < 0.001) for the first slope and β = 0.59 (*SE* = 0.11, *p* < 0.001) for the second slope. Therefore, we concluded that, again, both slopes are useful for predicting follow-up treatment outcomes (CAPS-5). However, relatively speaking, it is the change in PTSD symptomatology during the second week that is more informative for predicting the follow-up treatment outcome (measured using the CAPS-5) compared to the change in the first week. When the second slope is omitted, the explained variance in ΔC is reduced to 28.3%, implying that it is the combination of all three growth components that is most useful for predicting the follow-up treatment outcomes. 

## 4. Discussion

This paper studied whether change in PTSD symptomatology during treatment predicts treatment outcome. This was assessed in an outpatient intensive treatment program for PTSD, in which Prolonged Exposure (PE), Eye Movement Desensitization and Reprocessing (EMDR), physical activity and psychoeducation were combined. Progress during the six-day treatment (three consecutive days for two consecutive weeks) was monitored by assessing symptoms of PTSD with an abbreviated PTSD self-report measure (8-item PCL). It was expected that early improvement in PTSD symptoms during treatment would be a predictor for treatment outcome at a four-week follow-up. Consistent with the expectation, the results indicate that a greater decline in self-reported PTSD symptoms during the complete treatment program is a predictor for a greater decline in PTSD symptoms at a four-week follow-up, as measured with self-rated and clinician-rated instruments. Additionally, it was expected that a greater decline in PTSD symptoms during the first half of treatment would predict overall treatment outcomes. The results show that a greater decline in self-reported PTSD symptoms during the first week of treatment, after completing two of the total six treatment days, was indeed a predictor for treatment outcome at four-week follow-up, as measured with self-rated and clinician-rated instruments. These findings are consistent with earlier findings which showed that early PTSD-symptom change was related to overall treatment outcome [27,28]. Interestingly, discrepant results have been found concerning the predictive value of the first and second treatment week. When using the self-report PCL-5 as the overall treatment outcome measure, the first treatment week has more predictive value, whereas when using the clinician-rated CAPS-5, the second week has more predictive value. Moreover, the results show that pre-treatment PTSD symptoms, as measured with the self-report 8-item PCL, do not predict treatment outcomes. This is an interesting finding because the findings of previous studies showed that pre-treatment PTSD symptoms were in fact related to treatment outcome [14,15]. 

Since the results of studies on the value of pre-treatment factors were inconclusive [11,12,13,14,15,17] this stresses the clinical importance of the need for more approaches to predict treatment outcome. Although the study awaits replication, the results imply that using a measure to monitor treatment progress and evaluate early progress could be valuable in decision-making (e.g., adjusting treatment based on treatment response). When a patient does not show a large improvement during the first few days, practitioners may decide to scale up treatment or change the type of evidence-based treatment provided, which is what guidelines recommend [50]. Doing so might prevent patients from continuing a treatment with little-to-no benefit. Although in this case, scaling up treatment by intensifying seems difficult considering the treatment program for the current sample is already an intensive one. Other remaining guideline suggestions include switching to other evidence-based treatments (e.g., cognitive processing therapy), pharmacotherapy or experimental treatments.

The current study has a number of limitations. One of the limitations is that there were a number of missing cases, which were mainly explained by patients missing or showing up too late for the follow-up measurements. This might give a distorted picture of the representation of the population. However, it is unclear if and how this would have influenced the outcome. One might argue that the one reason why patients did not show up or showed up too late for the follow-up measurement is because they were unsatisfied with treatment and have seen little improvement in their symptoms. On the other hand, patients who do not show symptoms may refuse to spend their time and effort for measurement since they already finished treatment and did not see any value in it. A further limitation is that the patient sample predominantly consisted of women, which might affect generalizability of the results. It is unclear why the sample had such a notable gender imbalance, however the results should be interpreted with this in mind. The size of the sample can be seen as a limitation, since it made it impossible to investigate if the presence of comorbid disorders could influence treatment progress.

A strength of the current study is that treatment outcome is measured using two different measurements: a clinician-rated instrument (CAPS-5) and a self-report measure (PCL-5). Although the CAPS-5 and PCL-5 correlate strongly [45], one study found contradictory results in prediction models using the CAPS [51] and PCL [52] as treatment outcome measures [15]. They found that lower baseline CAPS scores were associated with better treatment outcomes, whereas higher baseline PCL scores were associated with better treatment outcomes. Using the clinician-rated CAPS-5 as well as the self-report PCL-5 to measure treatment outcome controls for these possibly ambiguous results.

Future research should be aimed at improving the reliability and generalizability of the results by replicating the current study in different cultures and in samples that vary in gender, age and other demographic variables. One could study if there is a difference in the results for trauma type and different types and intensities of treatment as well. Since the results were found in an intensive treatment program including PE and EMDR, the study should be replicated in non-intensive treatment programs and other types of treatments, even though there are indications that these results are generalizable to non-intensive treatment programs with other types of treatment [27,28]. Another recommendation for future research is to explore possible adaptations in intensive treatment programs for patients who show little response, since it is yet unclear which adaptations can be made. Therefore, future research should first be focused on investigating relevant processes during treatment that could influence outcomes in the current treatment program. The current treatment consists of several components, and one should also identify which treatment components are responsible for treatment progress, and also in whom. It is useful to know that the absence of response during treatment indicates less beneficial overall treatment outcome. The next step should be investigating what is causing patients to show little or no response, or even deterioration. As mentioned before, factors like therapeutic alliance, trauma-related belief change, between-session habituation and SUD scores during the first PE or EMDR session have been shown to be related to treatment outcome [18,19,20,21,22,23,24]. These are possible relevant factors during treatment that could explain why some patients show little reduction in PTSD symptoms during treatment. It might be useful to identify which factors (known and unknown) are responsible for less beneficial treatment response in the current study. This could be helpful in investigating how treatment could be enhanced for the patients who are poorly responding. Another reason why it is useful to identify which processes are responsible for less beneficial treatment response is that the predictive value of the current study is based on means and the results are not necessarily applicable to every patient. This poses an ethical dilemma for clinical use because practitioners would have to make a decision for the individual to change treatment (intensity) based on those means. 

In conclusion, the present study indicates that a greater decline in PTSD symptoms during the course of an intensive treatment program is a predictor for greater treatment outcome at a four-week follow-up. This prediction can also be made using the progress measured during the first treatment week, after completing two of the six treatment days. Pre-treatment PTSD symptoms had no predictive value for treatment outcome at the four-week follow-up. Being able to predict treatment outcomes using the progress measured during treatment shows a large potential for clinical use. Future research should mainly be focused on replicating the current results and improving the reliability and generalizability of the results. A next step would be investigating the factors responsible for poorer treatment responses.

## Figures and Tables

**Table 1:** Treatment program.

Activity	Duration (Minutes)
Pre-treatment Measurement (only on the first day)	45
Prolonged Exposure	90
Short Break	15
Physical Activity (yoga, exercises, running)	60
Lunch Break	30–45
EMDR	90
Short Break	15
Psycho-education	60
Measurements (Last treatment day)	45

Note. The measurements conducted on the last treatment day were not used in the current study.

**Table 2:** Descriptive data for the follow-up treatment outcomes and the daily 8-item PCL.

Variable	*N*	*M*	*SD*	*Min.*	*Max.*
ΔP	89	−22.05 ^1^	19.61	−69 ^2^	+12 ^2^
ΔC	99	−17.29	15.47	−53 ^2^	+14 ^2^
8-item PCL–Day 1	107	22.49	35.54	5	32
8-item PCL–Day 2	105	20.60	40.64	2	32
8-item PCL–Day 3	102	19.52	48.45	1	32
8-item PCL–Day 4	106	19.29	45.09	0	32
8-item PCL–Day 5	102	18.08	48.35	1	32
8-item PCL–Day 6	96	17.37	55.25	1	31

^1^ The sum of ΔP and the mean follow-up PCL-5 score does not exactly equal the mean pre-treatment PCL-5 score. This is due to 14 patients who are included in the pre-treatment score but are missing at the 4-week follow-up measurement and are therefore not included in the mean PCL-5 at 4-week follow-up and in ΔP. ^2^ ΔP and ΔC represent a change in PTSD symptomatology. Therefore, the column *Min.* actually represents the *greatest* observed decrease in symptomatology, and the column *Max*. the *smallest* observed decrease (in fact, an increase) in symptomatology.

## Data Availability

The data presented in this study are available on request from the corresponding author.

## Supplementary Materials

The following are available online at https://jeroendmulder.github.io/predicting-PTSD-using-LGCM, Full details on the different candidate models considered, their prediction performance, and model fit.
